# Unique and shared risk factors for early childhood victimisation and polyvictimisation in a Brazilian population-based birth cohort

**DOI:** 10.1016/j.lana.2024.100715

**Published:** 2024-03-13

**Authors:** Romina Buffarini, Carolina V.N. Coll, Michelle Degli Esposti, Joseph Murray

**Affiliations:** a Post-Graduate Program in Epidemiology, Federal University of Pelotas, RS, Brazil; b Human Development and Violence Research Centre (DOVE), Federal University of Pelotas, RS, Brazil

**Keywords:** Violence against children, Polyvictimisation, Risk factors, Brazil

## Abstract

**Background:**

Identifying modifiable risk factors for child victimisation and polyvictimisation (exposure to multiple types of victimisation) is critical for informing prevention efforts, yet little evidence is available in low- and middle-income countries. The authors aimed to estimate the prevalence of child victimisation and polyvictimisation, and examine unique and shared risk factors in a population-based cohort in Southern Brazil.

**Methods:**

Lifetime child victimisation was based on maternal report when children were aged 4 years old (N∼3900) and included five types of victimisation (conventional crime, child maltreatment, peer/sibling victimisation, sexual victimisation, and witnessing/indirect victimisation) and polyvictimisation. Based on a socioecological model, possible risk factors were examined in four levels: community, maternal and family, parent, and child.

**Findings:**

Conventional crime and peer/sibling victimisation were the most common types of victimisation (46.0 and 46.5%, respectively), followed by witnessing/indirect victimisation (27.0%), and child maltreatment (11.3%). Sexual victimisation had the lowest prevalence (1.4%). One in 10 (10.1%) children experienced polyvictimisation. In general, boys had higher victimisation rates than girls. There were few risk factors related only to specific types of victimisation (e.g., child disability was uniquely associated with child maltreatment and peer/sibling victimisation). Instead, most risk factors were shared across nearly all victimisation types and also associated with polyvictimisation. These shared risk factors were: violent neighbourhood and low social cohesion, maternal adverse childhood experiences, younger maternal age, parental antisocial behaviour, intimate partner violence against mothers, and maternal depression.

**Interpretation:**

These findings reveal a general pattern of accumulative risk effects for different types of victimisation and polyvictimisation, rather than unique risk profiles in children aged four year Prevention efforts should target risk factors at multiple levels (e.g.,: community, maternal and family and parent) during early childhood.

**Funding:**

10.13039/100010269Wellcome Trust grant 10735_Z_18_Z.

**Research in context**

**Evidence before this study**

Studies investigating risk factors for varied types of child victimisation such as peer/sibling violence, crime, and different types of abuse are scarce. Existing research typically focusses on specific types of victimisation (e.g., maltreatment) and is highly skewed towards studies in high-income countries, with very little data on polyvictimisation, especially among young children. The authors found only one population-based study in low- and middle-income countries on risk factors for different victimisation types and polyvictimisation, conducted with children aged 13–17 years in Cambodia, Kenya, Malawi, Nigeria, Tanzania and Haiti. There was substantial heterogeneity in risk factors across these countries, underscoring the importance of country-specific research to understand children's experiences of violence.

**Added value of this study**

We investigated the prevalence of varied types of child victimisation and polyvictimisation in a population-based Brazilian birth cohort, and explored possible common and unique modifiable risk factors across four levels of the socioecological model. The findings add to the scarce evidence from low- and middle-income settings overall, and particularly among young children. Given the limited evidence on population prevalence of, and risk factors for, multiple types of child victimisation in Brazil, this study highlights the need for targeted strategies to protect vulnerable children at the local and national level.

**Implications of all the available evidence**

Overall, the evidence suggests that risk factors for different types of child victimisation tend to be shared. Parent characteristics (antisocial behaviour and intimate partner violence), maternal and family factors (maternal adverse childhood experiences), and community features (e.g., low neighbourhood cohesion) were all robustly associated with almost all victimisation types. This suggests that an accumulation of various risk factors across different levels of the socioecological model, especially in the maternal and family and community contexts, places children at risk for varied types of victimisation. Possible interventions might prioritise targeting children exposed to multiple risk factors in early childhood to protect them from violence in multiple forms.

## Introduction

Violence against children is a major public health and human rights problem that affects more than 1 billion children every year around the world.^1^ Its impacts on the child, family, and society are pervasive. Children who are victimised face immediate risks (e.g., serious injury and trauma), as well as longer-term consequences persisting into adulthood (e.g., poor physical and mental health, unemployment, and premature death) and across generations.^2^^,^^3^ Child victimisation, defined as harm to the child's health cause by acts of human violence,^4^ can take many different forms, including maltreatment by caregivers, peer bullying, sexual abuse, and exposure to neighbourhood violence and crime, and rarely occurs in isolation.^5^ Children exposed to multiple types of victimisation (“polyvictims”) tend to experience more serious victimisations than other child victims and are at greatest risk of detrimental biopsychosocial impacts across the lifespan.^6^, ^7^, ^8^, ^9^, ^10^

Child victimisation has multiple determinants, shaped by a complex interplay of individual, family, social and environmental factors.^11^^,^^12^ Identifying modifiable risk factors across different levels of the socioecological model is critical to inform prevention efforts.^13^ Existing research on early childhood victimisation typically focusses on specific types of child maltreatment and is highly skewed towards studies in high-income countries.^11^^,^^14^^,^^15^ In a systematic review of risk factors for physical, emotional, and sexual violence against children in low- and middle-income countries. No specific patterns of risks were identified for these different outcomes; the heterogeneity in the definitions of violence and risk factors examined was highlighted.^16^ At the moment of the study literature revision, that occurred between January and June of 2023, the authors found that research on risk factors for overlapping forms of childhood victimisation (polyvictimisation) has generally been limited to consideration of few victimisation types (e.g., physical, sexual and emotional abuse)^17^ and fewer studies have examined risk factors in general population samples (cf. war-affected settings)^8^^,^^17^ and among young children.^13^ Notably the authors found only one study on risk factors for child polyvictimisation in low and middle-income countries. That study used data from the Violence Against Children Surveys, conducted in six countries (Cambodia, Kenya, Malawi, Nigeria, Tanzania and Haiti), and examined risk factors for emotional, physical and sexual victimisation, and polyvictimisation, among 13–17-year-olds.^17^ There was substantial heterogeneity in risk factors across countries, underscoring the importance of country-specific research to understand children's experience of violence in each context.

In Brazil, published literature is constrained to cases that have been officially reported to the police, judiciary, or health services,^18^, ^19^, ^20^, ^21^ making comparisons challenging. Additionally, this evidence is limited to certain types of victimization -such as neglect or sexual violence, and is characterised by a poor report quality.^19^^,^^22^ At a national level, the most recent data, as per police records and public security authorities, showed that from 2016 to 2019, approximately 800 children aged 0–9 years victims of violent deaths.^22^ In addition, in the age group between 0 and 4 years of age, nearly 90% of the cases were perpetrated by individuals known to the victims. Still, the available evidence regarding the prevalence of various forms of child victimization in the general population, as well as the associated risk factors, is currently limited. As a result, policymakers and practitioners lack insight on whether there are unique or common patterns of risk factors for children exposed to specific and/or multiple types of victimisation, which is critical for designing targeted strategies for protecting vulnerable children. In the current study, the authors aimed to address this gap in evidence by estimating the prevalence of different types of victimisation and polyvictimisation experienced during early childhood, and examining potential unique and shared risk factors in a large, population-based, birth cohort study in southern Brazil. The authors hypothesised that the different forms of violence, typically studied in separate literature, would share common risk factors.

## Methods

### Study participants

We analysed data from the 2015 Pelotas (Brazil) Birth Cohort Study. Pelotas is a city in southern Brazil, with around 340,000 inhabitants. All children delivered in hospitals in Pelotas between 1 January and 31 December 2015, and whose mother lived in the urban area of the city, were eligible for the cohort study. Over 99% of children born in Pelotas are delivered in hospitals. From the 4333 eligible live births, 4275 were assessed at delivery, equivalent to a response rate of 98.7%. All children and their mothers were invited to participate in follow-up assessments at 3, 12 and 24 months and 4 years, with response rates varying between 99.0% and 95.3%. Additional information about the 2015 Pelotas Birth Cohort Study is available elsewhere.^23^

### Outcomes

Child victimisation was assessed using the Portuguese version of the Juvenile Victimisation Questionnaire, 2nd edition, Screener Sum Version, Caregiver Lifetime Form (JVQ-R2).^24^^,^^25^ JVQ has been adapted and validated for use in Brazil.^26^ The questionnaire was administered in confidential interviews, which were conducted by trained female interviewers in a research centre with mothers or primary caregivers at the 4-year follow-up. Psychological support was available when positive responses were given. The JVQ includes five modules which capture different types of victimisation: conventional crime, child maltreatment, peer/sibling victimisation, sexual victimisation, and witnessing/indirect victimisation.^27^ Each module contains between four and nine question items (describing specific types of victimization within the module) and is scored positively if at least one of its constituent items is scored “yes” (Table S1). Of all 34 questions in the JVQ-R2, one (on dating violence) is used only with adolescents, yielding a total of 33 items for young children. Following previous studies,^28^^,^^29^ polyvictims were defined as children experiencing the most (top 10%) numbers of types of victimisation. This was operationalised by summing the number of positive responses to all 33 items (Fig. S1) and defining children with scores of 6 or more as polyvictims. All polyvictims experienced victimisation in at least two domains (Table S2) (conventional crime, child maltreatment, peer/sibling victimisation, sexual victimisation, and witnessing/indirect victimisation) in this sample. Between polyvictims, 15.9% experienced two domains, more than a half scored positive in three domains and about a third scored positive in four domains, 3% of polyvictims belonged to the five victimisation domains (Table S2).

### Possible risk factors

**Fig. 1:**
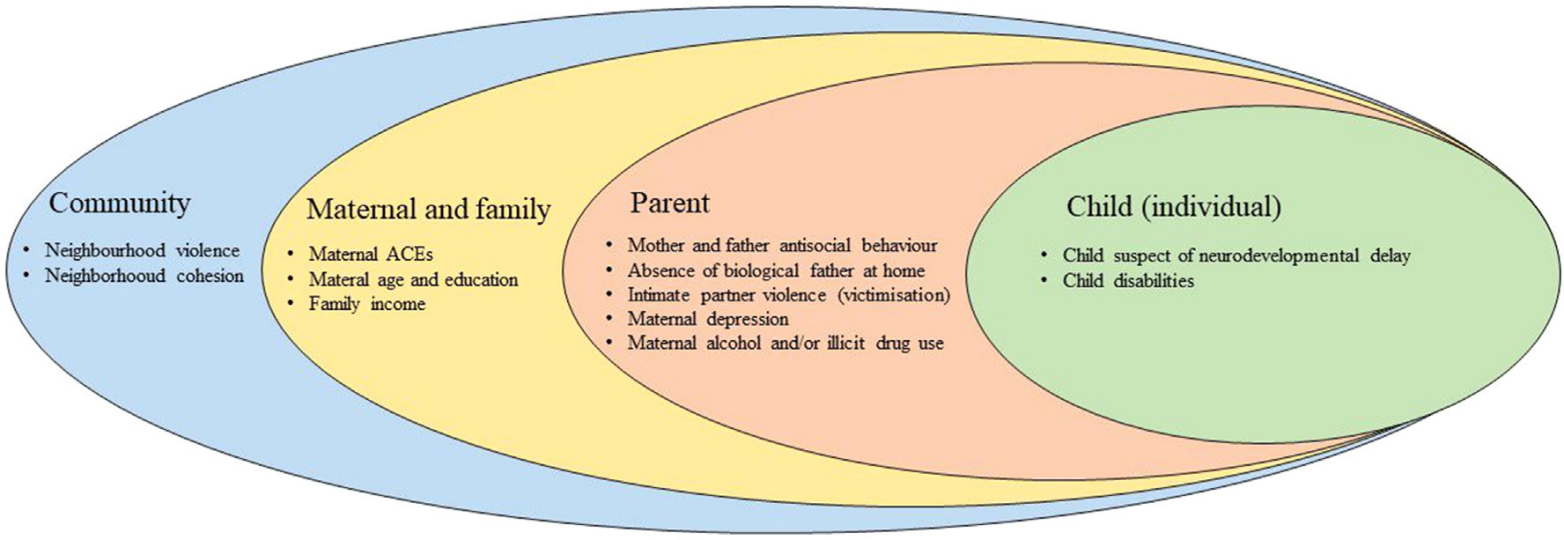
**Four-level social-ecological model**.

Possible risk factors were chosen *a priori* based on previous literature^11^^,^^14^^,^^15^^,^^17^ - carried out between January and June of 2023, and organized according to a four-levels in a socio-ecological model: child, parent, maternal and family, and community levels (Fig. 1).

### Child characteristics

Child characteristics included measures of neurological development and disabilities. To measure child development, the Oxford Neurodevelopment Assessment (Ox-NDA); was administered at age 12 months^30^ directly with the cohort child, and via interviews with mothers/caregivers. Suspected global neurodevelopmental delay (yes/no) was defined as children in the lowest 10th percentile. Child disabilities (yes/no) at age 4 years was defined by whether the cohort child had one or more of the following conditions: Down's Syndrome, autism spectrum disorders, epilepsy, cerebral palsy or mental retardation visual impairment, hearing disorder, intellectual disability, language disorders, and/or muscular dystrophy, reported on by the mother.

### Parent characteristics

Maternal depression was measured using the Edinburgh Postnatal Depression Scale (EPDS)^31^ at the 24-month follow-up and defined as having a score of 13 or more points. The following maternal and paternal characteristics were assessed in interviews with mothers/caregivers when children were aged 4 years: whether the biological or social father lived at home (excluding adoptive fathers, n = 24), parental antisocial behaviour, intimate partner violence (IPV) against the mother in the past 12 months,^32^ maternal drug and alcohol use in the three months prior to interview (Alcohol, Smoking and Substance Involvement Screening Test; ASSIST).^33^ Father and mother antisocial behaviour was evaluated using the Antisocial Personality Module of the Mini International Neuropsychiatric Interview (MINI),^34^ responded to by the mother. For the current analyses, the authors created an antisocial behaviour total score by summing five of the six (yes/no) questions on antisocial behaviour, excluding a sixth question on domestic violence (which was assessed separately), to test the extent to which parental general antisocial behaviours are a risk factor for child victimisation. Scores were dichotomised so that 1 or more represented antisocial behaviour.

### Maternal and family characteristics

Low family income (bottom two quintiles), low maternal education (<9 years schooling years) and young maternal age (<20 years at birth), were measured during the perinatal assessment. Maternal adverse childhood experiences up to age 18 years (ACEs), measured at age 4-year follow-up. Maternal ACEs were collected using a shortened version of the World Health Organization (WHO) ACE-IQ questionnaire,^35^ including nine types of adversities (emotional abuse, physical abuse, sexual abuse, violence against household members, living with substance abusers, living with household members who were mentally ill or suicidal, living with household members who were imprisoned, parents died or divorced). First, all adversities were summed to produce a total ACEs score ranging from 0 to 9, and then categorized into two groups: 0–3 and 4 or more (as a conventional cut-off point).^36^

### Community

Community social cohesion and danger in the neighbourhood were measured at the age 4-year follow-up. Neighbourhood cohesion was measured using five questions scored 0–3 (strongly agree to strongly disagree) about social trust, connectedness, and solidarity in the neighbourhood.^37^ The score was summed (higher numbers indicating lower levels of cohesion), and then dichotomised whereby 11 or more points indicated a neighbourhood with low cohesion. Neighbourhood violence was assessed using four questions about the frequency of violent acts in the neighbourhood in the last 6 months: fights with weapons, fights between gangs, robbery, and sexual violence. Possible responses, that ranged from never (0) to often (3), were summed,^38^ and violent neighbourhood was defined as scores of 8 or more.

### Ethics

The cohort study was approved by the Ethics Committee of the School of Physical Education, Federal University of Pelotas (CAAE registration number: 26746414.5.0000.5313) and psychosocial assessments measured at 4-year follow-up, were approved by the Ethics Committee of the Faculty of Medicine, Federal University of Pelotas (CAAE registration number: 03837318.6.0000.5317). Written informed consent was obtained from parents or guardians at each visit. Psychological support was available when positive responses regarding child victimisation were given.

### Analyses

The prevalence of risk factors and child victimisation were first described, stratified by sex. Prevalence ratios (PR) and 95% Confidence Intervals (CIs) for the unadjusted and adjusted associations between risk factors and child victimisation were estimated using Poisson regression with robust variance. Throughout, possible risk factors were grouped into four levels according to the socioecological model shown in Fig. 1.

In adjusted analyses modelling, a nine-level hierarchical model was adopted to avoid adjusting for possible mediating variables (Fig. S2).^39^ In each level estimates were adjusted for risk factors in previous levels, as well as other risk factors in the same level, except for variables in the 8th and 9th levels which were not adjusted for each other due to possible bidirectional associations. As such, variables from the first level (violent neighbourhood and neighbourhood cohesion) were entered simultaneously in the model, then maternal ACEs was included, keeping variables from level 1 and so on. For the 8th and 9th levels, separate models were estimated for each of the possible risk factors. For example, for the 8th level, IPV (model 8a), maternal depression (model 8b) and maternal alcohol and/or drugs (model 8c) were performed in three separate models, each of them, adjusted for precedent levels (1–7). All models also adjusted for child sex and age at time of assessment.

We guarded against inflated false positive (type I errors) from multiple testing by performing the analyses on the entire sample, and then applying Benjamini-Hochberg adjustment.^40^

Analyses were carried out in STATA 16.1 (StataCorp, College Station, USA).

### Role of funding source

The funder of the study had no role in the study design, data collection, data analysis, data interpretation, or writing of the report.

## Results

The analytical sample comprised 3993 participants with complete information on victimisation, representing 93.4% (3993/4275) of the original participants in the cohort recruited at birth and 99.6% (3933/4010) of participants at the 4-year follow-up. Half of sample were boys (50.6%).

The prevalence of risk factors ranged from 4.4% for maternal alcohol and substance use to 40.1% for low family income (Table 1). Maternal and family risk factors were generally the most prevalent in the sample, including 39.5% of mothers experiencing four or more ACEs up to 18 years of age, while the prevalence of child risk factors such as disability (20.2%) and others such as violent neighbourhood (8%) were lower. There were no statistically significant differences between the proportion of possible risk factors between sex, except for child disabilities that were higher in boys than in girls (24.3% versus 16.1%, p < 0.0001).

**Table 1:** Sample characteristics.

Possible risk factors	n (%)			p-value^a^
	Total (n = 3993)	Boys (n = 2023)	Girls (n = 1970)	
**Community characteristics**				
Violent neighbourhood	318/3979 (8.0)	163/2015 (8.1)	155/1964 (7.9)	0.86
Low neighbourhood cohesion	458/3976 (11.5)	237/2013 (11.8)	221/1963 (11.3)	0.62
**Maternal and family characteristics**				
Maternal ACEs (4+)	1567/3969 (39.5)	799/2011 (39.7)	768/1958 (39.2)	0.75
Young maternal age (<20 years)	581/3992 (14.6)	297/2022 (14.7)	284/1970 (14.4)	0.82
Low maternal education (<9 years)	1384/3992 (34.7)	714/2022 (35.3)	670/1970 (34.0)	0.41
Low family income (1st & 2nd quintiles)	1599/3991 (40.1)	803/2022 (39.7)	796/1969 (40.4)	0.65
**Parent characteristics and behaviours**				
Father antisocial behaviour	848/3837 (22.1)	436/1934 (22.5)	412/1903 (21.7)	0.51
Mother antisocial behaviour	773/3967 (19.5)	379/2009 (18.9)	394/1958 (20.1)	0.34
Absence of biological father at home	1146/3962 (28.9)	576/2006 (28.7)	570/1956 (29.1)	0.78
Intimate partner violence	853/3757 (22.7)	428/1899 (22.5)	425/1858 (22.9)	0.82
Maternal depression	436/3915 (11.1)	224/1981 (11.3)	212/1934 (11.0)	0.76
Maternal use of alcohol and/or illicit drugs	176/3973 (4.4)	89/2012 (4.4)	87/1961 (4.4)	0.99
**Child characteristics**				
Suspected neurodevelopment delay	358/3513 (10.2)	185/1772 (10.4)	173/1741 (9.9)	0.66
Child disabilities	808/3993 (20.2)	491/2023 (24.3)	317/1970 (16.1)	<0.0001

ACEs, Adverse childhood experiences.

Father antisocial behaviours and child suspect of neurodevelopment delay had the highest missing values (4 and 7%, respectively).

a Fisher's exact test for the difference between boys and girls.

Lifetime child victimisation information was collected at the mean age of 3.8 (SD = 0.2) years. Table 2 shows the prevalence of each type of victimisation by domain and polyvictimisation. Conventional crime and peer/sibling victimisation had the highest prevalence (46.0 and 46.5%, respectively), while sexual victimisation was the lowest (1.4%). One in 10 children experienced polyvictimisation (10.1%). Boys experienced a higher prevalence of victimisation types and polyvictimisation, except for witnessing/indirect and sexual victimisation where there were no significant sex differences. Table S1 shows the prevalence of individual types of victimisation experienced by children in the cohort. The most common forms of victimisation experienced were: robbery (26.1%) in the conventional crime domain, emotional abuse by a caregiver (7.9%) in the maltreatment domain, physical intimidation (29.6%) in the peer/sibling victimisation domain, sexual assault by peer/sibling (0.7%) in the sexual victimisation domain, and exposure to random shootings or riots (7.4%) in the witnessing/indirect domain. Remarkably 194 children (4.9%) in the cohort had someone close (friend, neighbour or any family member) who was murdered during the child's lifetime.

**Table 2:** Prevalence of specific types of victimisation and polyvictimisation in the sample, stratified by sex.

Modules of victimisation	Prevalence: % (95% CI)			p-value^a^
	All	Boys	Girls	
Conventional crime	46.0 (44.5; 47.6)	48.5 (46.3; 50.7)	43.5 (41.3; 45.7)	0.01
Child maltreatment	11.3 (10.3; 12.3)	12.6 (11.2; 14.1)	10.0 (8.7; 11.3)	0.01
Peer/sibling victimisation	46.5 (45.0; 48.1)	49.6 (47.4; 51.8)	43.4 (41.2; 45.6)	<0.0001
Sexual victimisation	1.4 (1.0; 1.8)	1.4 (1.0; 2.0)	1.3 (0.9; 1.9)	0.89
Witnessing/indirect victimisation	26.9 (25.5; 28.3)	26.5 (24.7; 28.5)	27.3 (25.4; 29.3)	0.06
Polyvictimisation	10.1 (9.2; 11.1)	11.3 (10.0; 12.7)	8.8 (7.7; 10.2)	0.01

Polyvictimisation defined as 6 or more positive questions on the JVQ-R2.

a Fisher's exact test for the difference between boys and girls.

Almost all possible risk factors were associated with conventional crime, child maltreatment, peer/sibling victimisation, witnessing/indirect victimisation, and polyvictimisation in the unadjusted associations (Table S3). Only maternal ACEs, parent-level characteristics (mother antisocial behaviour, absence of father at home and maternal IPV) and low socioeconomic level were associated with sexual victimisation. There was no significant association between child neurodevelopment and victimisation of any type (Table S3).

**Fig. 2:**
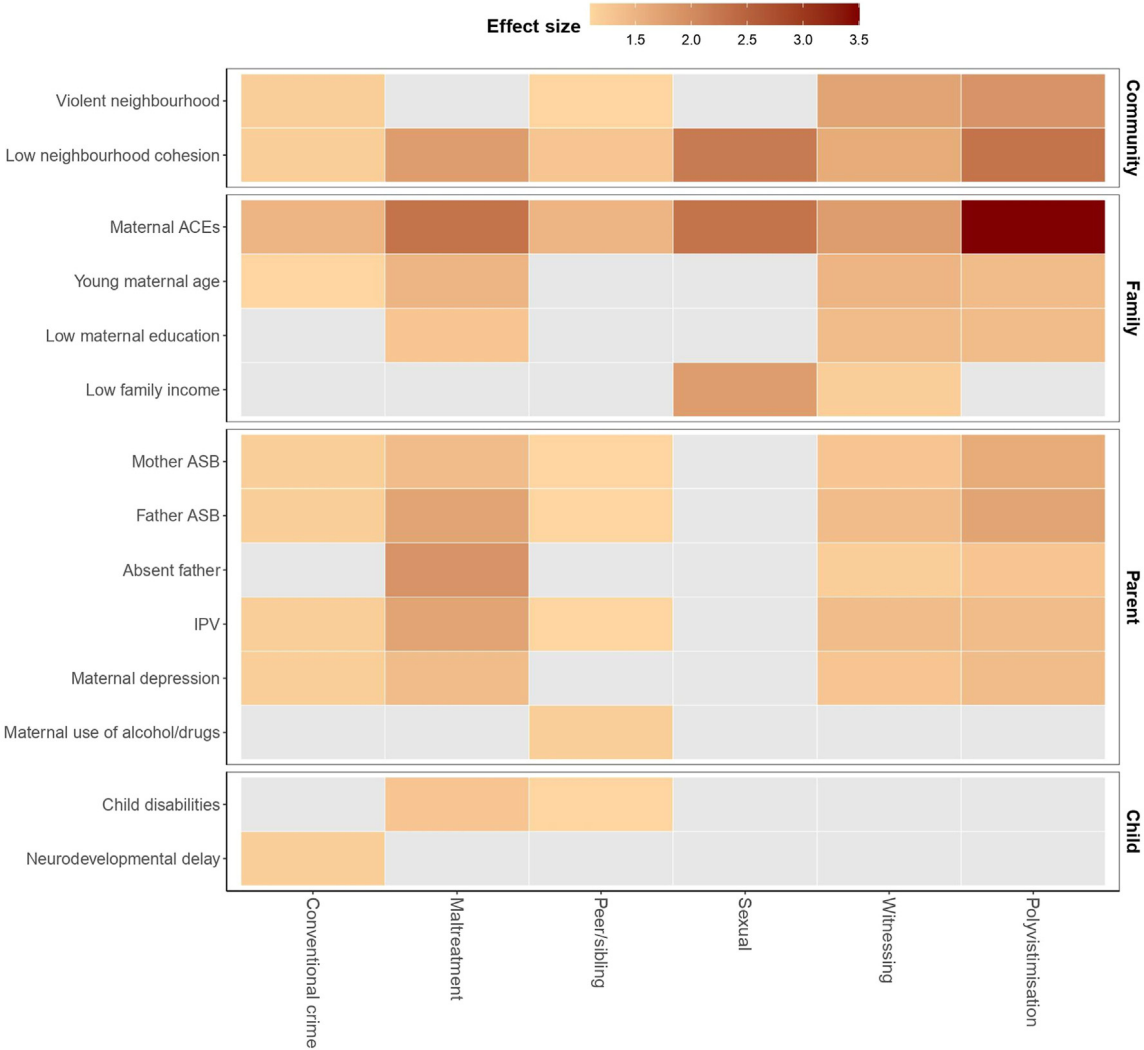
**Heatmap of adjusted associations between victimisation outcomes and risk factors in the 2015 Pelotas Birth Cohort, Brazil. ∗Cells in grey are not significant associations**.

In adjusted models (Table 3, Fig. 2), there were several shared risk factors for multiple different types of victimisation, particularly in terms of parent, maternal and family and community characteristics. For example, maternal ACEs were associated with all domains of victimisation (PRs ranging from 1.47 to 3.45; p-values <0.0001), as was low neighbourhood cohesion (PRs ranging from 1.23 to 2.271; p-values all <0.05). IPV and parental antisocial behaviour were associated with all victimisation types other than sexual (Fig. 2). Young maternal age also associated with all victimisation types except for conventional crime, peer/sibling and sexual victimisation, while depression was only not associated to peer/sibling and sexual victimisation. Polyvictimisation showed robust associations with these same parent and maternal and family risk factors. Children's own characteristics were not consistently associated with individual victimisation domains or polyvictimisation. Instead, child disability was specifically associated with child maltreatment and peer/sibling victimisation. Other risk factors with specific associations included low maternal education and low family income, which were only associated with witnessing/indirect victimisation.

**Table 3:** Adjusted associations between victimisation outcomes and risk factors in the 2015 Pelotas Birth Cohort, Brazil.

Levels	Possible risk factors	PR (95% CI)					
		Conventional crime	Child maltreatment	Peer/sibling victimisation	Sexual victimisation	Witnessing/indirect victimisation	Polyvictimisation
**Community characteristics**							
1	Violent neighbourhood	1.17 (1.06; 1.31)^b^	1.12 (0.83; 1.50)	1.13 (1.02; 1.26)^a^	1.68 (0.78; 3.64)	1.69 (1.48; 1.94)^c^	1.87 (1.46; 2.39)^c^
1	Low neighbourhood cohesion	1.23 (1.13; 1.35)^c^	1.82 (1.46; 2.26)^c^	1.27 (1.17; 1.39)^c^	2.23 (1.17; 4.24)^a^	1.64 (1.45; 1.85)^c^	2.27 (1.83; 2.81)^c^
**Maternal and family characteristics**							
2	Maternal ACEs (>4)	1.50 (1.41; 1.61)^c^	2.28 (1.90; 2.74)	1.47 (1.38; 1.58)^c^	2.35 (1.37; 4.02)^b^	1.76 (1.59; 1.95)^c^	3.45 (2.80; 4.27)^c^
3	Young maternal age (<20 years)	1.09 (1.00; 1.19)	1.52 (1.24; 1.86)	1.02 (0.93; 1.11)	0.91 (0.44; 1.88)	1.52 (1.35; 1.70)^c^	1.42 (1.15; 1.75)^c^
4	Low maternal education (<9 years)	1.02 (0.95; 1.09)	1.29 (1.08; 1.55)	0.95 (0.88; 1.02)	1.06 (0.60; 1.88)	1.41 (1.27; 1.57)^c^	1.41 (1.17; 1.70)^c^
5	Low family income (1st & 2nd quintiles)	1.00 (0.93; 1.07)	1.14 (0.94; 1.39)	1.00 (0.93; 1.08)	1.81 (1.03; 3.17)^a^	1.22 (1.09; 1.36)^c^	1.20 (0.98; 1.46)
**Parent characteristics and behaviours**							
6	Mother antisocial behaviour	1.19 (1.10; 1.29)^c^	1.38 (1.12; 1.71)^b^	1.15 (1.06; 1.25)^c^	0.91 (0.47; 1.77)	1.29 (1.15; 1.46)^c^	1.59 (1.29; 1.96)^c^
6	Father antisocial behaviour	1.20 (1.11; 1.30)^c^	1.65 (1.34; 2.04)^c^	1.12 (1.04; 1.21)^b^	1.71 (0.97; 3.03)	1.43 (1.27; 1.60)^c^	1.73 (1.41; 2.14)^c^
7	Absence of biological father at home	1.04 (0.96; 1.12)	1.91 (1.56; 2.33)^c^	0.99 (0.92; 1.08)	0.64 (0.30; 1.37)	1.19 (1.06; 1.33)^b^	1.29 (1.06; 1.57)^a^
8a	Intimate partner violence	1.23 (1.14; 1.33)^c^	1.72 (1.40; 2.12)^c^	1.11 (1.03; 1.20)^a^	1.66 (0.88; 3.13)	1.43 (1.27; 1.60)^c^	1.42 (1.16; 1.76)^b^
8b	Maternal depression	1.20 (1.10; 1.32)^c^	1.45 (1.14; 1.83)^b^	1.03 (0.93; 1.14)	0.95 (0.44; 2.07)	1.29 (1.13; 1.48)^c^	1.43 (1.13; 1.82)^b^
8c	Maternal use of alcohol and/or illicit drugs	1.04 (0.90; 1.20)	1.11 (0.79; 1.54)	1.16 (1.02; 1.31)^a^	0.33 (0.05; 2.28)	1.09 (0.89; 1.32)	1.35 (0.99; 1.83)
**Child characteristics**							
9a	Child disabilities	1.07 (0.99; 1.16)	1.28 (1.04; 1.58)^a^	1.12 (1.03; 1.21)^b^	1.05 (0.55; 1.99)	1.02 (0.90; 1.16)	1.11 (0.89; 1.38)
9b	Child suspect of neurodevelopment delay	1.15 (1.03; 1.29)^a^	0.97 (0.68; 1.38)	1.06 (0.95; 1.19)	0.46 (0.11; 1.93)	1.04 (0.87; 1.24)	1.01 (0.70; 1.45)

^a^p < 0.05 ^b^p < 0.01 ^c^p < 0.0001.

p-values correspond to Wald test.

Note on column Levels: variables are adjusted for variables in all previous levels as well as other variables in the same level—except for variables in the 8th and 9th levels, which are not adjusted for each other.

Adjusted for child sex.

After applying the Benjamini-Hochberg correction, results did not change, with exception of low family income that lost association with sexual victimisation.

## Discussion

In this study, the authors investigated the prevalence of varied types of child victimisation and polyvictimisation in a Brazilian population-based birth cohort, and possible common and unique risk factors. The overall prevalence of child victimisation was high, with almost half of this general population sample experiencing peer/sibling victimisation or conventional crime, such as robbery. Multiple types of victimisation commonly co-occurred, and around 1 in 10 children had six or more types of violence experiences, therefore, were defined as polyvictims. The most frequent types of victimisation (conventional crime and peer/sibling victimisation, as well as polyvictimisation) were more prevalent among boys than girls. Few risk factors were unique to specific types of victimisation (one example was child disability that uniquely associated with child maltreatment and peer/sibling victimisation). Instead, parent, maternal and family, and wider community risk factors were generally shared across different victimisation types, as well as polyvictimisation. This suggests that an accumulation of common risk factors place children at increased risk of different types of violent victimisation in a middle-size city in Southern Brazil.

The high prevalence of conventional crime and exposure to witnessing violence may be specific to the location of this Latin American sample. Brazil is known to have high rates of crime and community violence^41^ and the southern city of Pelotas—where this study was conducted—mirrors high national rates.^42^ Our finding that almost half of children had experienced conventional crime victimisation by the age of 4 years suggests that infants and young children are not protected from community violence, and evidence from other studies shows even proximity to severe violence in the community has significant impact on children's development.^43^ It should be noted that several of the most frequent “conventional crime” items reported in this study could have been interpreted in relation to events that actually happened between peers/siblings (such as using force to take things the child was carrying or wearing). However, other types of “crime” victimisation, such as “attacked with an object or weapon” (5%) and kidnapped (1%) were also reported, and exposure to serious adult violence was not uncommon in this sample: murder of someone close to the child experienced by 5%; shootings or other serious violence witnessed by 7%. The high prevalence of peer/sibling victimisation in this age group, however, echoes findings from high-income settings, which identify peer/sibling victimisation as a common type of victimisation among young children with an earlier onset compared to other types, such as maltreatment and sexual victimisation.^44^ The setting and young age of our study sample may thus shape which victimisation types were identified to be most common, raising some questions about the comparability of different victimisations. However, these concerns do not invalidate the importance of assessing the wide range of experiences that child victimisation implies.^45^ Future studies should continue to monitor the developmental epidemiology of child victimisation to advance understanding of the scope and characteristics of violent victimisation in this and other low-resource settings. As such, even though peer/sibling assaults are seen as less serious victimisation events in young children, more studies are needed to assess the possible traumatizing effect of this type of experiences. We identified that around 10% of children aged 4-years old experienced six or more victimisation experiences, highlighting how frequently and early in development child victimisation can co-occur. While these rates of polyvictimisation were substantially lower than estimates for children aged between 13 and 17 in other low- and middle-income countries (e.g., Haiti, Nigeria, Cambodia), adolescents would be expected to have higher lifetime rates given the significantly longer window for possible exposure.^17^ More comparable rates of polyvictimisation among a similar age group (2–5 years old) in the US places 8% of children having experienced seven or more types of violence.^6^ In line with previous studies from both low- and high-income settings, the authors also found that boys were at increased risk of polyvictimisation than girls.^6^^,^^17^ Cross-national research can help to identify which characteristics of victimisation are context-specific (e.g., prevalence) and which may be universal (e.g., the role of gender), and thus inform targeted prevention and response strategies for those children at greatest risk.

Overall, there was strong evidence for common risk factors for different types of child victimisation and polyvictimisation. Characteristics relating to parents (antisocial behaviour and IPV), the maternal and family (maternal ACEs), and community (e.g., low neighbourhood cohesion) were all robustly associated with almost all victimisation types. This suggests that there are few unique risk factors for specific types of child victimisation. Rather, it is an accumulation of various risk types across different levels of the socioecological model, especially in the maternal and family and community settings (rather than child characteristics), that places young children at risk of *any* victimisation.^11^^,^^13^^,^^17^ Notably, maternal ACEs emerged as the most robust and consistent risk factor for any and all victimisation types, adding to the growing evidence of the importance of breaking the intergenerational transmission of childhood adversity.^46^^,^^47^ The authors also found that risk factors relating to the children themselves were weakly associated with victimisation types, suggesting that child characteristics before 4 years old play a minimal role in shaping a child's risk profile. Instead, research and interventions should focus on investigating and intervening at the level of the maternal and family and community to build an evidence base for population-wide prevention strategies.

While our results suggested common rather than unique risk factors for child victimisation, the authors also note some specific patterns of associations for victimisation types. For example, a violent neighbourhood was associated with all victimisation types except for child maltreatment and sexual victimisation. This may reflect the nature of maltreatment being more determined by the home environment, such as characteristics relating to the parents and maternal and family directly. In this context, the absence of biological father was associated with child maltreatment, witnessing/indirect victimisation, and polyvictimisation. Previous studies have consistently identified the absence of a biological father as a risk factor for child maltreatment,^48^ aligning with evidence indicating that social fathers tend to perpetrate maltreatment more than biological fathers.^49^^,^^50^ Additionally, the absence of a biological father has been reported as a risk factor for violence against women,^48^ possibly contributing to its association with witnessing/indirect victimisation. On the other hand, peer/sibling victimisation was associated with child characteristics; specifically children with disabilities were at higher risk of being bullied.^51^ Although targeting common risk factors may be key to effective prevention, it is also important to acknowledge and address risk factors related to specific victimisation types to shed light on the mechanisms leading to heightened vulnerability to specific outcomes.^14^

This study is not without limitations. Despite the large population-based sample with high response rates, some differences were observed between those with complete information and those who were losses (Table S4). Losses were higher in the extreme maternal education groups, and higher family income group, leading to possible bias estimations. Also, there is a possible lack of power for detecting certain associations, especially for sexual victimisation—which was experienced by less than 1.5% of the sample. As a result, not significant associations for sexual victimisation may be attributed to a lack of power, rather than a lack of association. Sexual victimisation evokes a social stigma and is often underreported, both in population-surveys^16^ and official reports,^22^ in make it challenging to get an accurate picture of how widespread sexual violence is. The authors also explored the possibility of collecting official records of child victimization, but the number of cases officially registered in Pelotas was extremely small. This suggests that relying on official data would likely result in almost all true cases being coded as false negatives. Therefore, the low prevalence observed in our study might reflect the complex difficulties involved in measuring the true extent of sexual violence within a population. Overall, reporting of child victimisation is plagued with methodological issues.^52^ In this sample, child victimisation is measured using maternal reports on the validated JVQ questionnaire,^25^ which is the most appropriate measure given the young age of the children.^52^ Nevertheless, reports should always be used with careful attention to some potential limitations. On one hand, child victimisation might be biased in both under and overestimation by maternal mental health. In general, mothers who are emotionally distressed tend to be less accurate reports of her own parenting. There is also a potential for social desirability bias regarding her own child's victimisations. On the other hand, some victimisation events may not have been known to the mother and/or response and recall biases may impact the reliability of our outcome measure.^52^ Furthermore, the definition of polyvictimisation varies both within and across instruments. While the authors followed the most common approach for the JVQ of classifying the top 10% of the sample as polyvictims (6 plus questions), in some studies assessing adolescents, this cut-off implied a more stringent criteria (e.g.,: 10 plus items).^29^^,^^53^^,^^54^ Since our sample was 4-year-old children who have a narrow exposure window, the authors chose a lower threshold, which is desirable when the purpose is identifying vulnerable children.^28^ However, findings could vary depending on different cut-off points used. Finally, it is necessary to interpret the results of this study considering the specific characteristics of single urban city cohort. These findings may not be applicable to the entire country of Brazil or rural areas, as the sample might not accurately represent the diverse socio-economic variations present nationwide.

In conclusion, this study details the prevalence of child victimisation and identifies common risk factors placing children from a Brazilian urban population-based cohort at increased risk of multiple types of victimisation. Our findings add to the scarce evidence from low- and middle-income settings and show that, even by 4 years of age, almost half of children have experienced at least one type of victimisation, most commonly conventional crime or peer/sibling victimisation. The authors also show that polyvictimisation among this young age group is concerningly prevalent, with around 1 and 10 children having experienced 6 or more different episodes of violence. While there is some evidence of unique risks for specific victimisation types, risk factors generally shared across them. Thus, the cumulative exposure across multiple levels of risk (parent, maternal and family, community) may be most important in determining risk. Our results suggest that population-wide interventions might consider prioritising the targeting multiple risk factors in early childhood in order to break the intergenerational transmission of adversity and violence.

## Contributors

RB conceived the study and designed the analysis plan with JM, CC and MDE. RB received the raw data. RB and MDE verified data. Data curation and analysis was undertaken by RB and MDE. All authors interpreted the data. RB, CC and MDE drafted the first draft of the manuscript. All authors made substantial contributions to the revision of the manuscript and had final decision to submit it.

## Data sharing statement

Applications to use the data can be made by contacting the researchers of the 2015 cohort (see http://www.epidemio-ufpel.org.br/site/content/faculty/ for a list of key faculty members) and completing the application form (http://www.epidemio-ufpel.org.br/site/content/studies/formularios.php). Researchers with successful applications will receive a dataset including the requested variables and unique participant IDs.

## Declaration of interest

We declare no competing interests.

## Appendix A Supplementary data

Supplemental material review clean

Abstract in Portuguese
