# Supplementary material for: Unique and shared risk factors for early childhood victimisation and polyvictimisation in a Brazilian population-based birth cohort
Source: Lancet Reg Health Am. 2024 Mar 13;32:100715. doi: 10.1016/j.lana.2024.100715 (PMC10950884; doi:10.1016/j.lana.2024.100715)
Supplement: Supplemental material review clean [file mmc1.docx]

**Supplementary material**

| **Table S1.** Prevalence of 33 questions of Screener Sum Version JVQ-R2 adapted for children aged 4 years | | | |
| --- | --- | --- | --- |
| **Modules** | **Item Question** | | **N (%)** |
| **Conventional crime** | Robbery | At any time in your child’s life, did anyone use force to take something away from your child that your child was carrying or wearing? | 1041 (26.1) |
|  | Personal Theft | At any time in your child’s life, did anyone steal something from your child and never give it back? Things like a backpack, money, watch, clothing, bike, stereo, or anything else? | 199 (5.0) |
|  | Vandalism | At any time in your child’s life, did anyone break or ruin any of your child’s things on purpose? | 968 (24.3) |
|  | Assault with weapon | Sometimes people are attacked with sticks, rocks, guns, knives, or other things that would hurt. At any time in your child’s life, did anyone hit or attack your child on purpose with an object or weapon? Somewhere like: at home, at school, at a store, in a car, on the street, or anywhere else? | 203 (5.1) |
|  | Assault without weapon | At any time in your child’s life, did anyone hit or attack your child without using an object or weapon? | 533 (13.4) |
|  | Attempted assault | At any time in your child’s life, did someone start to attack your child, but for some reason, it didn’t happen? For example, someone helped your child or your child got away? | 238 (6.0) |
|  | Threatened assault | At any time in your child’s life, did someone threaten to hurt your child when your child thought they might really do it? | 168 (4.2) |
|  | Kidnapping | At any time in your child’s life, did anyone try to kidnap your child? | 37 (0.9) |
|  | Bias attack | At any time in your child’s life, has your child been hit or attacked because of your child’s skin color, religion, or where your family comes from? Because of a physical problem your child has? Or because someone said your child was gay? | 34 (0.9) |
| **Child Maltreatment** | Corporal/physical punishment | Not including spanking on your child’s bottom, at any time in your child’s life, did a grown-up in your child’s life hit, beat, kick, or physically hurt your child in any way? | 73 (1.8) |
|  | Emotional abuse | At any time in your child’s life, did your child get scared or feel really bad because grown-ups in your child’s life called your child names, said mean things to your child, or said they didn’t want your child? | 316 (7.9) |
|  | Neglect | When someone is neglected, it means that the grown-ups in their life didn’t take care of them the way they should. They might not get them enough food, take them to the doctor when they are sick, or make sure they have a safe place to stay. At any time in your child’s life, was your child neglected? | 83 (2.1) |
|  | Family abduction | Sometimes a family fights over where a child should live. At any time in your child’s life, did a parent take, keep, or hide your child to stop your child from being with another parent? | 87 (2.2) |
| **Peer/sibling victimisation** | Gang of group assault | Sometimes groups of kids or gangs attack people. At any time in your child’s life, did a group of kids or a gang hit, jump, or attack your child? | 35 (0.9) |
|  | Peer/sibling assault | At any time in your child’s life, did any kid, even a brother or sister, hit your child? Somewhere like: at home, at school, out playing, in a store, or anywhere else? | 1083 (27.1) |
|  | Nonsexual Genital Assault | At any time in your child’s life, did any kids try to hurt your child’s private parts on purpose by hitting or kicking your child there? | 45 (1.1) |
|  | Physical Intimidation by peers | At any time in your child’s life, did any kids, even a brother or sister, pick on your child by chasing your child or grabbing your child or by making your child do something your child didn’t want to do? | 1181 (29.6) |
|  | Relational aggression by peers | At any time in your child’s life, did your child get scared or feel really bad because kids were calling your child names, saying mean things to your child, or saying they didn’t want your child around? | 498 (12.5) |
| **Sexual victimisation** | Sexual assault by known adult | At any time in your child’s life, did a grown-up your child knows touch your child’s private parts when they shouldn’t have or make your child touch their private parts? Or did a grown-up your child knows force your child to have sex? | 10 (0.3) |
|  | Sexual assault by unknown adult | At any time in your child’s life, did a grown-up your child did not know touch your child’s private parts when they shouldn’t have, make your child touch their private parts or force your child to have sex? | - |
|  | Sexual assault by peer/sibling | Now think about other kids, from school or even a brother or sister. At any time in your child’s life, did another child or teen make your child do sexual things? | 26 (0.7) |
|  | Forced sex | At any time in your child’s life, did anyone try to force your child to have sex; that is, sexual intercourse of any kind, even if it didn’t happen? | 7 (0.2) |
|  | Flashing/ sexual exposure | At any time in your child’s life, did anyone make your child look at their private parts by using force or surprise, or by “flashing” your child? | 15 (0.4) |
|  | Verbal sexual harassment | At any time in your child’s life, did anyone hurt your child’s feelings by saying or writing something sexual about your child or your child’s body? | 6 (0.2) |
|  | Statutory rape & sexual misconduct | At any time in your child’s life, did your child do sexual things with anyone 18 or older, even things your child both wanted? | 2 (0.1) |
| **Witnessing/indirect victimisation** | Witness to domestic violence | At any time in your child’s life, did your child SEE a parent get pushed, slapped, hit, punched, or beat up by another parent, or their boyfriend or girlfriend? | 254 (6.4) |
|  | Witness to parent assault of sibling | At any time in your child’s life, did your child SEE a parent hit, beat, kick, or physically hurt his or her brothers or sisters, not including a spanking on the bottom? | 98 (2.5) |
|  | Witness to assault with weapon | At any time in your child’s life, in real life, did your child SEE anyone get attacked on purpose WITH a stick, rock, gun, knife, or other thing that would hurt? Somewhere like: at home, at school, at a store, in a car, on the street, or anywhere else? | 236 (5.9) |
|  | Witness to assault without weapon | At any time in your child’s life, in real life, did your child SEE anyone get attacked or hit on purpose WITHOUT using a stick, rock, gun, knife, or something that would hurt? | 312 (7.8) |
|  | Burglary of family household | At any time in your child’s life, did anyone steal some thing from your child’s house that belongs to your child’s family or someone your child lives with? Things like a TV, stereo, car, or anything else? | 270 (6.8) |
|  | Murder of family member or friend | At any time in your child’s life, was anyone close to your child murdered, like a friend, neighbor or someone in your child’s family? | 194 (4.9) |
|  | Exposure to random shootings or riots | At any time in your child’s life, was your child in any place in real life where your child could see or hear people being shot, bombs going off, or street riots? | 301 (7.4) |
|  | Exposure to war or ethnic conflict | At any time in your child’s life, was your child in the middle of a war where your child could hear real fighting with guns or bombs? | 59 (1.5) |

**Fig S1**. Number of positive items of JVQ-R2 in children aged 4 years. 2015 Pelotas Birth Cohort.

**Table S2**. Polyvictims according to number of victimization modules (N=402)

| Modules score* | N (%) |
| --- | --- |
| 2 | 64 (15.9) |
| 3 | 208 (51.6) |
| 4 | 118 (29.5) |
| 5 | 12 (3.0) |
| Total polyvictims | 402 (100) |

*First column refers to the number of modules in which polyvictims are included


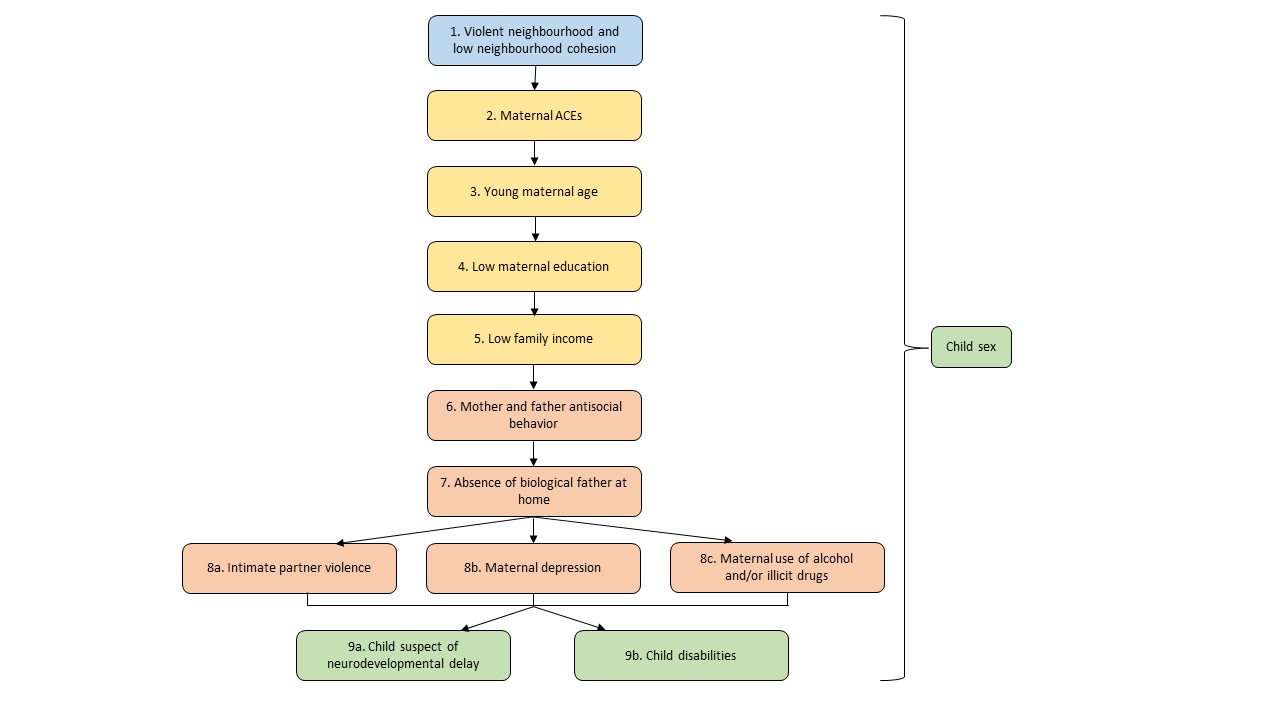


**Fig S2.** Hierarchical model in nine levels for adjusted analyses

| **Table S3.** Unadjusted associations between victimization outcomes and risk factors in the 2015 Pelotas Birth Cohort, Brazil. | | | | | | | |
| --- | --- | --- | --- | --- | --- | --- | --- |
|  | **Hypothesized risk factors** | **PR (95% CI)** | | | | | |
|  |  | **Conventional crime** | **Child maltreatment** | **Peer/sibling victimisation** | **Sex victimisation** | **Witnessing/indirect victimisation** | **Polyvictimisation** |
| **Community characteristics** | |  |  |  |  |  |  |
|  | Violent neighbourhood | 1.2 (1.1;1.4)*** | 1.3 (1.0;1.7) | 1.2 (1.1;1.3)** | 2.0 (1.0;4.2) | 1.9 (1.6;2.1)*** | 2.3 (1.8;2.9)*** |
|  | Low neighbourhood cohesion | 1.3 (1.2;1.4)*** | 1.8 (1.5;2;3)*** | 1.3 (1.2;1.4)*** | 2.4 (1.3;4;5)** | 1.8 (1.6;2.0)*** | 2.5 (2.0;3.1)*** |
| **Family characteristics** | |  |  |  |  |  |  |
|  | Maternal ACEs (> 4) | 1.5 (1.4;1.6)*** | 2.4 (2.0;2.9)*** | 1.5 (1.4;1.6)*** | 2.6 (1.5;4.5)** | 1.9 (1.7;2.1)*** | 3.9 (3.1;4.7)*** |
|  | Young maternal age (<20 years) | 1.1 (1.0;1.2)** | 1.7 (1.4;2.1)*** | 1.1 (1.0;1.2) | 1.0 (0.5;2.2) | 1.6 (1.4;1.8)*** | 1.6 (1.3;2.0)*** |
|  | Low maternal education (<9 years) | 1.1 (1.0;1.2)** | 1.6 (1.4;2.0)*** | 1.0 (1.0;1.1) | 1.3 (0.8;2.2) | 1.7 (1.6;1.9)*** | 1.9 (1.6;2.3)*** |
|  | Low family income (1^st^ & 2^nd^ quintiles) | 1.1 (1.0;1.2)* | 1.5 (1.3;1.8)*** | 1.0 (1.0;1.1) | 2.0 (1.2;3.4)* | 1.6 (1.4;1.7)*** | 1.7 (1.4;2.1)*** |
| **Parent characteristics and behaviours** | |  |  |  |  |  |  |
|  | Mother antisocial behavior | 1.4 (1.3;1.5)*** | 2.3 (1.9;2.8)*** | 1.3 (1.2;1.4)*** | 2.2 (1.3;3.8)** | 1.9 (1.7;2.1)*** | 2.9 (2.4;3.5)*** |
|  | Father antisocial behavior | 1.4 (1.3;1.5)*** | 2.2 (1.9;2.7)*** | 1.3 (1.2;1.4)*** | 1.4 (0.8;2.6) | 1.9 (1.7;2.1)*** | 2.9 (2.4;3.4)*** |
|  | Absence of biologial father at home | 1.2 (1.1; 1.2)*** | 2.4 (2.1; 3.0)*** | 1.1 (1.0; 1.1) | 0.9 (0.5; 1.7) | 1.6 (1.4; 1.8)*** | 1.9 (1.6; 2.3)*** |
|  | Intimate partner violence | 1.5 (1.4;1.6)*** | 2.6 (2.2;3.1)*** | 1.3 (1.2;1.4)*** | 2.3 (1.3;4.0)** | 2.0 (1.8;2.2)*** | 2.6 (2.2;3.2)*** |
|  | Maternal depression | 1.4 (1.2;1.5)*** | 2.0 (1.6;2.4)*** | 1.1(1.0;1.2)* | 1.4 (0.7;3.0) | 1.7 (1.5;2.0)*** | 2.3 (1.8;2.8)*** |
|  | Maternal use of alcohol and/or ilicit drugs | 1.3 (1.1;1.4)*** | 1.7 (1.2;2.4)** | 1.3 (1.2;1.5)*** | 0.4 (0.1;2.9) | 1.6 (1.4;1.9)*** | 2.2 (1.7;3.0)*** |
| **Child characteristics** | |  |  |  |  |  |  |
|  | Child disabilities | 1.2 (1.1;1.3)*** | 1.6 (1.4;2.0)*** | 1.2 (1.1;1.3)*** | 1.1(0.6;2.1) | 1.2 (1.1;1.4)*** | 1.5 (1.3;1.9)*** |
|  | Child suspect of neurodevelopment delay | 1.1 (1.0;1.2) | 1.0 (0.7;1.3) | 1.0 (0.9;1.2) | 0.8 (0.3;2.3) | 1.0 (0.9;1.2) | 1.0 (0.7;1.4) |
| *p<.05 **p<.01 ***p<.0001 | | | | | | | |
| p-values correspond to Wald test | | | | | | | |

**Table S4.** Comparison between analytical sample and losses to follow-up or non-response participants at 4y follow-up in the 2015 Pelotas Birth Cohort (N=4275).

| Characteristics | Losses/ non-response*  (N=282) | Complete information  (N=3993) |
| --- | --- | --- |
|  | % | |
| Sex | p=0.85 | |
| Boys | 50.0 | 50.7 |
| Girls | 50.0 | 49.3 |
| Maternal formal education (years) | p<0.0001 | |
| 0 to 4 | 14.5 | 8.8 |
| 5 to 8 | 21.6 | 25.9 |
| 9 to 11 | 22.7 | 34.9 |
| 12 or more | 41.1 | 30.4 |
| Family income (quintiles) | p=0.01 | |
| 1 (poorer) | 22.3 | 19.6 |
| 2 | 15.3 | 20.5 |
| 3 | 17.4 | 20.2 |
| 4 | 17.0 | 20.3 |
| 5 (richest) | 28.0 | 19.5 |
| *Losses at 4y follow-up = 265, non-responses at 4y follow-up= 17.  p-values correspond to Chi-Squared test | | |
