# Supplementary material for: Unique and shared risk factors for early childhood victimisation and polyvictimisation in a Brazilian population-based birth cohort
Source: Lancet Reg Health Am. 2024 Mar 13;32:100715. doi: 10.1016/j.lana.2024.100715 (PMC10950884; doi:10.1016/j.lana.2024.100715)
Supplement: Abstract in Portuguese [file mmc2.docx]

**Editor note:** *This translation in Portuguese was submitted by the authors and we reproduce it as supplied. It has not been peer reviewed. Our editorial processes have only been applied to the original abstract in English, which should serve as reference for this manuscript.*

**Resumo**

**Contexto:** Identificar fatores de risco modificáveis para vitimização infantil e polivitimização (exposição a múltiplos tipos de vitimização) é crucial para orientar esforços de prevenção, porém, há pouca evidência disponível em países de baixa e média renda. Os autores visaram estimar a prevalência de vitimização infantil e polivitimização, e examinar fatores de risco únicos e compartilhados em uma coorte de base populacional no Sul do Brasil.

**Métodos:** A vitimização infantil ao longo da vida foi baseada no relato materno quando as crianças tinham 4 anos de idade (N ~ 3900) e incluiu cinco tipos de vitimização (crime convencional, maus-tratos infantis, vitimização por colegas/irmãos, vitimização sexual e testemunhar/vitimização indireta) e polivitimização. Com base em um modelo socioecológico, possíveis fatores de risco foram examinados em quatro níveis: comunidade, maternos e familiares, parental e criança.

**Resultados:** Crime convencional e vitimização por colegas/irmãos foram os tipos mais comuns de vitimização (46,0% e 46,5%, respectivamente), seguidos por testemunhar/vitimização indireta (27,0%) e maus-tratos infantis (11,3%). A vitimização sexual teve a menor prevalência (1,4%). Uma em cada 10 (10,1%) crianças experimentou polivitimização. Em geral, os meninos apresentaram taxas de vitimização mais altas do que as meninas. Houve poucos fatores de risco relacionados apenas a tipos específicos de vitimização (por exemplo, a deficiência infantil foi associada exclusivamente a maus-tratos infantis e vitimização por colegas/irmãos). Em vez disso, a maioria dos fatores de risco foi compartilhada entre quase todos os tipos de vitimização e também associada à polivitimização. Esses fatores de risco compartilhados incluíram: bairro violento e baixa coesão social, experiências adversas na infância materna, idade materna mais jovem, comportamento antissocial dos pais, violência do parceiro íntimo contra as mães e depressão materna.

**Interpretação:** Os achados revelam um padrão geral de efeitos cumulativos de risco para diferentes tipos de vitimização e polivitimização, em vez de perfis de risco exclusivos.

**Financiamento:** Bolsa da Wellcome Trust 10735_Z_18_Z
